# Revision of the Neotropical *Neuratelia* Rondani (Diptera, Mycetophilidae, Sciophilinae): two new species, a new combination, and a new synonym

**DOI:** 10.3897/zookeys.861.32835

**Published:** 2019-07-08

**Authors:** Carolina Henao-Sepúlveda, Marta Wolff, Dalton de Souza Amorim

**Affiliations:** 1 Grupo de Entomología, Universidad de Antioquia, Calle 67 # 53-108, Medellín, Colombia Universidad de Antioquia Medellín Colombia; 2 Departamento de Biologia, Faculdade de Filosofia, Ciências e Letras de Riberão Preto, Universidade de São Paulo, Avenida Bandeirantes 3900, 14040-901, São Paulo, Brazil Universidade de São Paulo São Paulo Brazil

**Keywords:** Andean ecosystem, biogeography, Neotropical diversity, taxonomy

## Abstract

We describe two new Neotropical species of *Neuratelia* Rondani from the high Central Andes of Colombia, *N.altoandina***sp. nov.** and *N.colombiana***sp. nov.** The holotype of *Eudicranaelegans* Lane actually is a species of *Neuratelia* and a new combination is proposed. Our examination of the holotype of *Neurateliasapaici* Lane from southeastern Brazil shows this species to be a synonym of *N.elegans* (Lane), which is formally proposed here. *Neurateliasapaici* is redescribed. The position of these three species within the genus is discussed. A key for the Neotropical species of *Neuratelia* is provided.

## Introduction

*Neuratelia* Rondani, 1856 is a clearly monophyletic genus which currently includes 31 species (16 Palaearctic, 13 Nearctic, one Oriental, and one Neotropical) (Kurina et al. 2015). In Borkent and Wheeler’s (2013) broad phylogenetic analysis of the Sciophilinae, *Neuratelia* was resolved as sister to a large clade that included all genera of the subfamily except *Acomoptera* Vockeroth, *Drepanocercus* Vockeroth, *Loicia* Vockeroth, *Paratinia* Mik, *Taxicnemis* Tonnoir and Edwards, *Aneura* Marshall, and *Phthinia* Winnertz. Søli’s (1997) mycetophilid phylogeny, which included 13 taxa of Sciophlinae, showed *Neuratelia* as basal to the clade including all other Sciophilinae except *Anaclileia* and *Phthinia*. The only divergence between both studies concerns *Anaclileia* Meunier, which in Borkent and Wheeler’s (2013) tree is placed inside the large clade sister to *Neuratelia*.

Søli et al. (2000) and Borkent and Wheeler (2013) characterized *Neuratelia* as a genus with a setose laterotergite, vein sc-r placed before the origin of Rs, C not produced beyond the apex of R_5_, R_5_ sinuous, presence of both medial and cubital forks, stem of M_1+2_ shorter than fork, M_1_ incomplete basally, and the origin of the anterior fork beyond that of the posterior fork.

*Neuratelia* is one of the rarest genera of Mycetophilidae in the Neotropical region. The catalogue of the family (Oliveira and Amorim 2014) includes a single species for the genus, *N.sapaici*, which is known only from the male holotype collected in 1947 at the Estação Biológica de Boracéia in the Atlantic Forest of the state of São Paulo, southern Brazil (Lane 1952). Despite intensive collecting of insects in this area over many decades, no other specimen of *Neuratelia* has been found so far.

Recent extensive collecting in the temperate environments of Colombia, mainly in the paramos of the high-Andean ecosystems, revealed a number of mycetophilid genera previously unknown for Colombia. This includes representatives of genera with Holarctic distributions, such as *Docosia* Winnertz and *Cordyla* Meigen (Oliveira and Amorim 2011; Kurina and Oliveira 2015), genera with southern temperate distribution, such as *Paraleia* Tonnoir and *Duretophragma* Borkent (shared mostly between Chile, Andean Argentina, and southern Brazil, sometimes with species in Australia), and the special case of the genus *Eumanota* Edwards (Manotinae), previously known only from the Oriental region (Amorim et al. 2018). Examining the holotype of *Eudicranaelegans* Lane (Lane 1948) we realize that it is a species of *Neuratelia* and a synonym of *Neurateliasapaici* Lane (Lane 1952).

In this paper, we describe two new species of *Neuratelia* from the paramo of the Central Andes of Colombia. We also propose a new combination for *Eudicranaelegans*, the synonymy of *N.sapaici* with *E.elegans*, and redescribe the species. A key for Neotropical species of *Neuratelia* is provided, and their taxonomic relationships are discussed.

## Materials and methods

**Figure 1.**
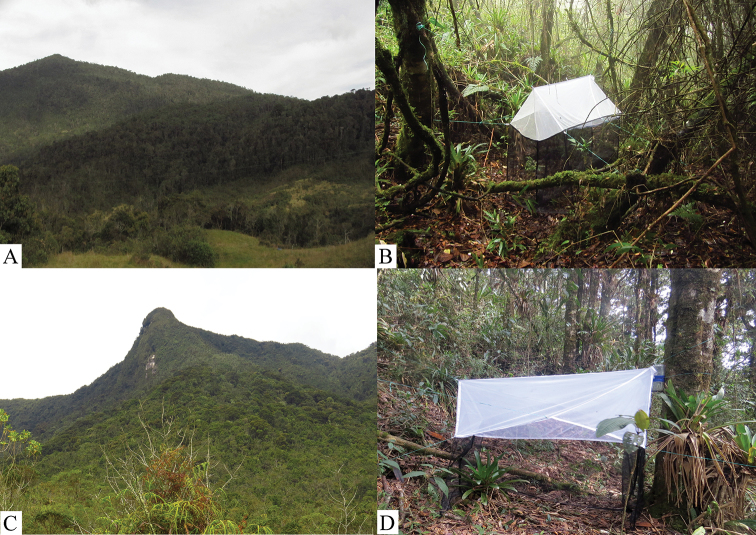
**A** Type locality of *Neurateliaaltoandina* sp. nov. (Holotype), landscape municipality of San José de la Montaña, paramo El Congo, Colombia **B** Malaise trap **C** type locality of *Neurateliacolombiana* sp. nov. (Holotype), landscape municipality of Sonsón, Norí mountain, paramo of Sonsón, Colombia **D** Malaise trap.

The Colombian specimens examined in this study are deposited in the Diptera collection of the Colección Entomológica Universidad de Antioquia (CEUA), at the Departmento de Antioquia, Medellin, Colombia. The specimens were collected between 2011 and 2014 with Malaise traps in the Central Andes in the Department of Antioquia, Colombia (Fig. 1A–D). The materials were originally preserved in 96% ethanol. One wing and the terminalia were separated from the rest of the body. Wings were mounted in permanent slides in Euparal. After removal, the terminalia were kept in KOH 10% for 12 h and then heated for 10 min, neutralized in acetic acid for 10 min, dehydrated in ethanol 70–90%, and preserved in a microvial in glycerine. The holotype of *N.elegans* is mounted on a pin, deposited at the Museu de Zoologia da Universidade de São Paulo (MZUSP), São Paulo, Brazil.

Photographs of the Colombian types were taken using a Moticam 3.0 megapixel DFC500 digital camera attached to an Olympus SZX7 stereomicroscope; the type of the Brazilian species was photographed with a Leica DC 500 camera coupled to a Leica M16 stereomicroscope. Photo stacking was performed using Helicon Focus v. 6.7.2 and edited with Adobe Photoshop CC 2017; photographs and illustrations of the terminalia were preparing using the U-DA Olympus drawing tube attached to an Olympus BX40 compound microscope, then vectorized with Illustrator CC 2017. Morphological terminology for head, thorax, pleural sclerites, and terminalia follows Søli (1997), Amorim and Oliveira (2008), and Kurina et al. (2015), while terminology of the wing venation follows Cumming and Wood (2017). For easy comparisons with other papers, we used Kurina et al.’s (2015) abbreviation system.

## Results

### Genus *Neuratelia* Rondani

#### Neuratelia

Taxon classificationAnimaliaDipteraMycetophilidae

Rondani 1856: 195.

##### Type species.

*Mycetophilanemoralis* Meigen (original designation).

##### Diagnosis.

(modified from Matile 1974; Søli et al. 2000; Sasakawa 2004; Borkent and Wheeler 2013). Vein R_5_ sinuous, sc-r placed basal to origin of Rs, C not produced beyond apex of R_5_, stem of M_1+2_ shorter than medial fork, base of M_1_ absent, origin of posterior fork basal to origin of anterior fork. Tibiae with distinct setae. Laterotergite and mediotergite setose.

### Key to Neotropical species of *Neuratelia*

**Table:** 

1	Scutum with a pair of dark lateral stripes and a pair of slender dorsocentral stripes (Fig. 3H); CuA gradually curving on apical third, reaching wing margin at an acute angle; CuP short, extending only half the length of CuA (Fig. 4B); terminalia with digitiform cerci (Figs 5F, 6D) (northeastern Colombia)	***N.colombiana* sp. nov.**
–	Scutum more or less homogeneously brown (Figs 3G, I); CuA strongly curved on apical third, reaching wing margin at angle of ~90°; CuP long, reaching the distal third of CuA (Figs 4A, C); terminalia with lobular cerci (Figs 5C, I, 6B, F)	**2**
2	Syngonocoxite fully covering entire ventral surface of terminalia (Figs 5A, 6A); dorsal branch of gonostylus digitiform; gonostylus with ventral branch digitiform (Figs 5B, 6B) (northwestern Colombia)	***N.altoandina* sp. nov.**
–	Syngonocoxite covering only anterior half terminalialia ventrally (Fig. 5G); gonostylus triangular, with a flat dorsal projection; gonostylus without ventral branch digitiform (Figs 5H, 6E) (southeastern Brazil)	***N.elegans* (Lane)**

#### Neuratelia altoandina sp. nov.

Taxon classificationAnimaliaDipteraMycetophilidae

http://zoobank.org/4918CB5F-E452-41BE-AB33-518E539E90F4

**Figure 2.**
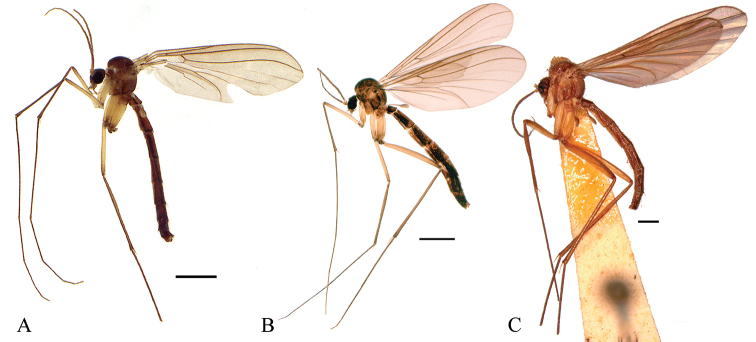
**A** Male habitus of *Neurateliaaltoandina* sp. nov. (holotype) **B** male habitus of *Neurateliacolombiana* sp. nov. (holotype) **C** male habitus of *Neurateliaelegans* (Lane) (holotype of *N.sapaici*). Scale bars: 1 mm.

**Figure 3.**
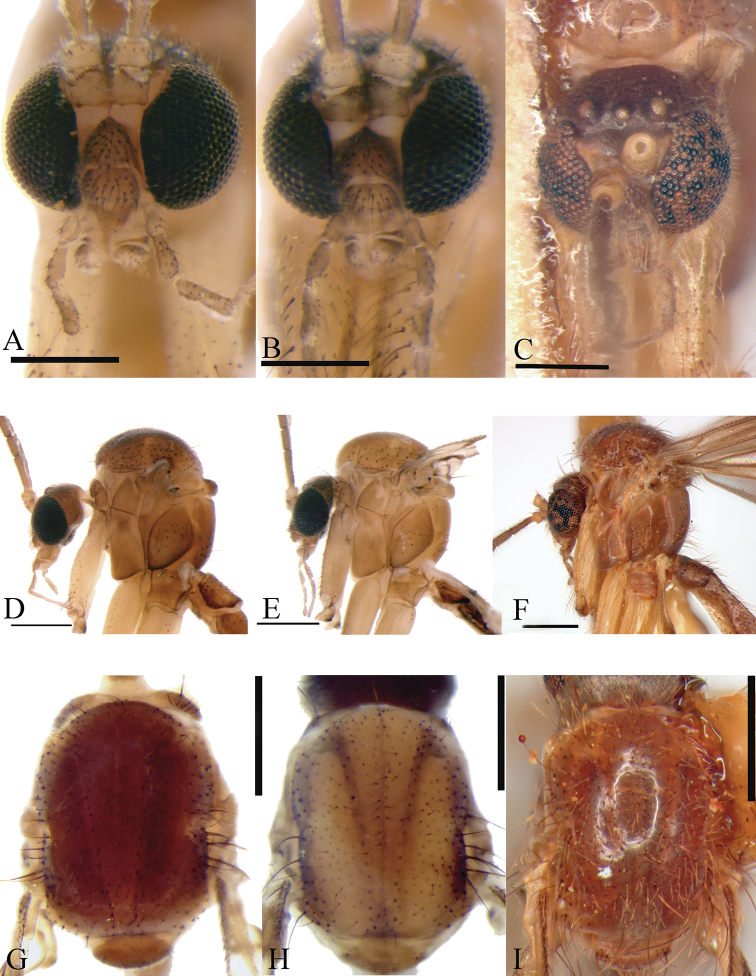
**A** Frontal head *Neurateliaaltoandina* sp. nov. (holotype) **B** frontal head *Neurateliacolombiana* sp. nov. (holotype) **C** frontal head *Neurateliaelegans* (Lane) (holotype of *N.sapaici*) **D** lateral thorax *Neurateliaaltoandina* sp. nov. (holotype) **E** lateral thorax *Neurateliacolombiana* sp. nov. (holotype) **F** lateral thorax *Neurateliaelegans* (Lane) (holotype of *N.sapaici*) **G** dorsal thorax *Neurateliaaltoandina* sp. nov. (holotype) **H** dorsal thorax *Neurateliacolombiana* sp. nov. (holotype) **I** dorsal thorax *Neurateliaelegans* (Lane) (holotype of *N.sapaici*). Scale bars: 0.25 mm.

**Figure 4.**
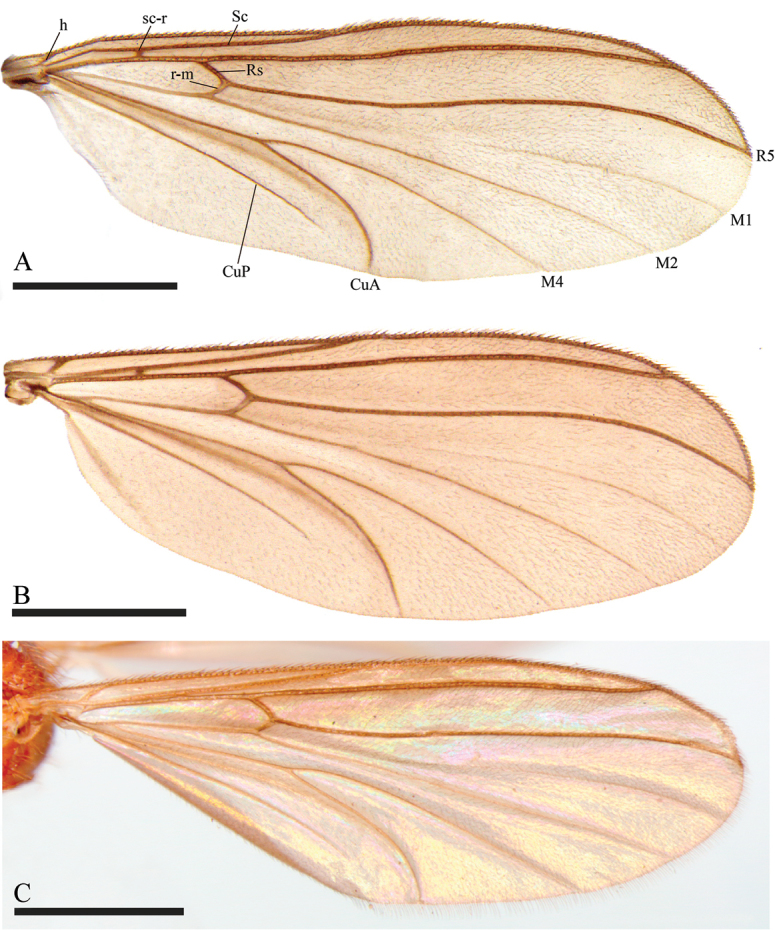
**A** Wing of *Neurateliaaltoandina* sp. nov. (holotype) **B** wing of *Neurateliacolombiana* sp. nov. (holotype) **C** Wing of *Neurateliaelegans* (Lane) (holotype of *N.sapaici*). Scale bars: 1 mm.

**Figure 5.**
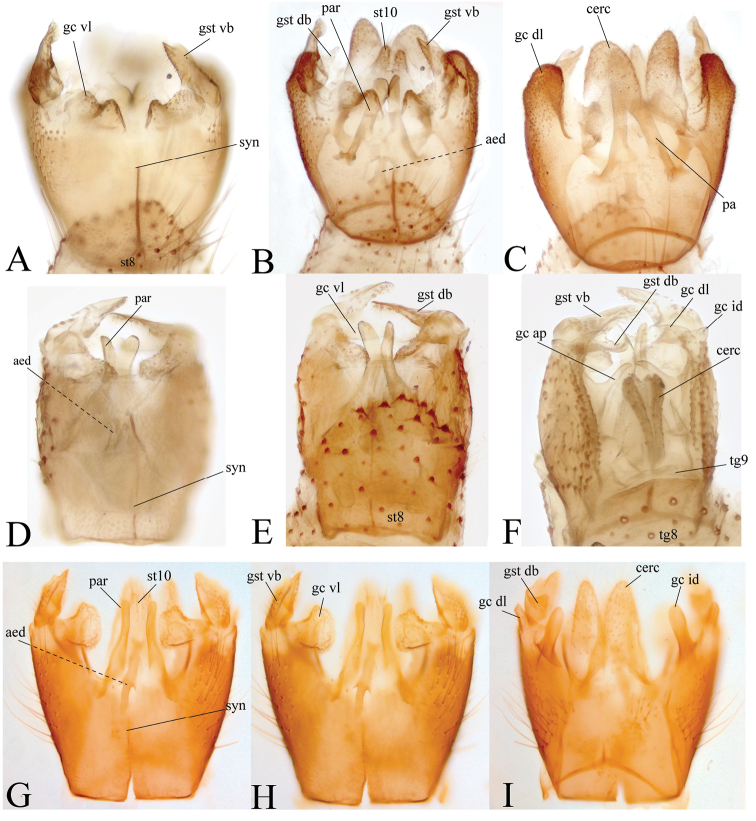
**A** Syngocoxite ventral view of the male terminalia of *Neurateliaaltoandina* sp. nov. (holotype) **B** ventral view **C** dorsal view **D** syngocoxite ventral view of male terminalia of *Neurateliacolombiana* sp. nov. (holotype) **E** ventral **F** dorsal view **G** syngocoxite ventral view of male terminalia of *Neurateliaelegans* (Lane) (holotype of *N.sapaici*) **H** ventral view **I** dorsal view.

**Figure 6.**
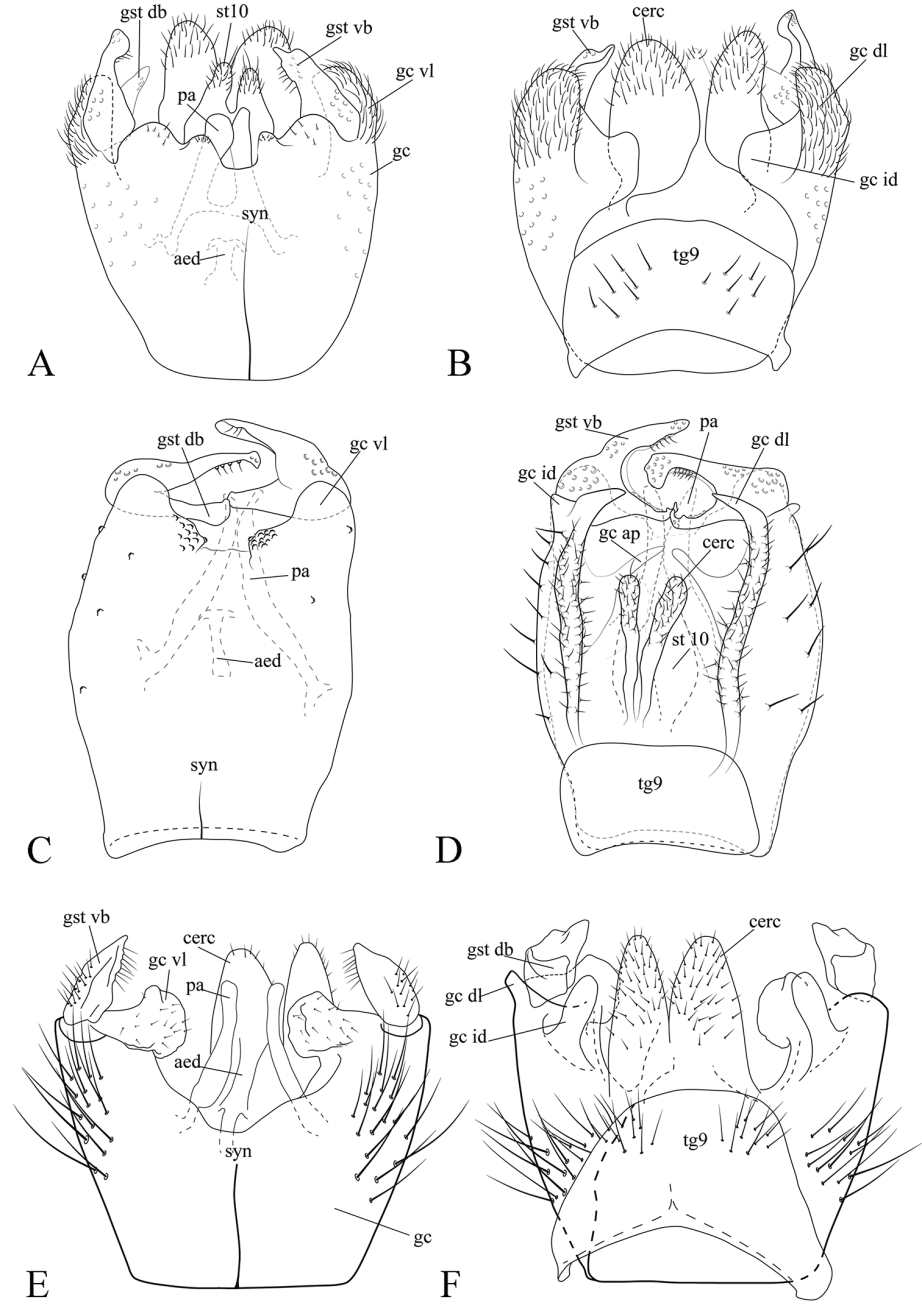
**A** Male terminalia illustrations of *Neurateliaaltoandina* sp. nov. (holotype) **B** ventral view **C** dorsal view **D** male terminalia illustrations of *Neurateliacolombiana* sp. nov. (holotype) **E** ventral view **F** dorsal view **G** male terminalia illustrations of *Neurateliaelegans* (Lane) (holotype of *N.sapaici*) **H** ventral view I dorsal view. Abbreviations: **aed** = aedeagus; **cerc** = cercus; **gc** = gonocoxite; **gc ap** = gonocoxal apodeme; **gc id** = gonocoxite inner dorsal projection; **gc dl** = gonocoxite dorso-apical lobe; **gc vl** = gonocoxite ventral lobe; **gst** = gonostylus, **gst db** = dorsal branch of gonostylus; **gst vb** = ventral branch of gonostylus; **syn** = syngocoxite; **par** = paremeres.

Figs 2A , 3A, D, G , 4A , 5A–C , 6A, B

##### Type locality.

Colombia, department of Antioquia, San José de la Montaña municipality, El Congo municipal rural settlement, paramo El Congo locality, 6°46.5651'N, 75°42.5701'W, alt. 3000 m a.s.l.; forest, L. Rios leg.

##### Type specimen.

Holotype male, wing mounted in Euparal on microscope slide, rest of body in alcohol 96%, genitalia in glycerine microvial. Original label: “ Colombia, Antioquia, San José de la Montaña, Vda. El Congo, páramo El Congo; 6°46'33.91"N, 75°43'34.21"W, 3000 m a.s.l.; forest, Malaise trap; 10–15 Sept. 2011; L. Ríos col.; CEUA 94078”.

##### Material examined.

**Holotype** ♂, Colombia, Department of Antioquia, San José de la Montaña municipality, El Congo municipal rural settlement, paramo El Congo locality; 6°46.551'N, 75°42.5701'W; alt. 3000 m a.s.l.; Malaise trap forest, 10–15 Sept. 2011; L. Rios leg., CEUA 94078.

##### Diagnosis.

Thorax brown, scutum with a pair of lighter longitudinal stripes medially. CuA with strong apical curve, reaching wing margin at an angle of about 90°, CuP long, ending at distal third of CuA. Syngonocoxites wide, fused medially, extending posteriorly almost to level of apical end of gonocoxites. Gonocoxite with a wide dorso-posterior lobular projection. Dorsal gonostylus shape like clamps, tapering apically.

##### Description.

**Male** (Fig. 2A). Body length, 5.8 mm. ***Head*** (Fig. 5A). Width 0.57 mm, height 0.35 mm. Vertex brown, with abundant brownish-yellow short setae. Three ocelli, mid ocellus smaller; lateral ocelli separated from eye margin by less than their diameter. Occiput chestnut brown. Ommatrichia abundant, short, yellowish. Scape, pedicel brownish yellow, cylindrical, scape slightly longer than pedicel, both with small brownish-yellow setae; 14 flagellomeres, mostly light brown, with scattered small dark setae; first flagellomere almost twice as long as second. Frons, clypeus brown, longer than wide, subtriangular; palpus with five palpomeres, light brown, apical palpomere twice as long as fourth. ***Thorax*** (Figs 3D, G). Mostly brown. Scutum with medial, light brown, triangular area, wide at anterior margin narrowing towards scutellum. A row of stronger setae present above wing; a single row of differentiated dorsocentrals. Scutellum with scattered smaller setae over disc, some longer setae along margin. Pleural sclerites mostly chestnut brown, katepisternum and laterotergite dark brown ventrally. Pleural membrane yellowish brown. Antepronotum with nine setae, proespisternum with three setae of different size. Proepimeron, anepisternum, katepisternum, mesepimeron, and metepisternum bare, laterotergite with about 20 dark large setae, mediotergite with 9 or 10 dark long setae laterally on the basal area. Halter pedicel yellowish, knob chestnut brown, setose. ***Legs.*** Coxae, femora yellow, tibia, tarsi brown. Foreleg tibia with ventral oval depression distally with abundant and irregularly distributed trichia; first tarsomere 1.5 times tibia length. Tibiae and tarsi with dark, short erect setae along their whole length. Tibial spurs 1:2:2, light brown, spurs as long as tibia apical width. Tarsal claws with large apical tooth, smaller basal tooth. ***Wing*** (Fig. 4A). Length, 5.0 mm, width, 2.0 mm. Membrane light brown, densely covered with macrotrichia, decumbent on all cells; veins brown. Sc complete, setose ventrally, reaching C well beyond base of Rs, almost at mid of the wing; sc-r present, bare, basal to the mid of Sc. C ending at apex of R_5_. R_1_ long, reaching C beyond apical fifth of wing. First sector of Rs oblique, setose ventrally, slightly longer than r-m; R_5_ sinuous, reaching C at wing apex; r-m bare, oblique. Medial and cubital veins complete, reaching wing margin. M_1+2_ stem shorter than anterior fork. M_1_ weak, obsolete basally. CuA strongly curved towards wing margin for apical third, reaching margin at an angle of about 90°. CuP long, reaching level of apical third of CuA. ***Abdomen.*** Segments chestnut brown, cylindrical, slender, brownish long setae covering tergites, sternites. Sternite 8 longer than wide, projecting medially, tergite 8 wider than long, also projecting medially. ***Terminalia*** (Figs 5A–C, 6A, B). Slightly wider than longer, gonocoxite ventral surfaces almost fused medially, forming a syngonocoxite with a ventral deep medial cleft, extending nearly to level of ventroapical margin of gonocoxite; gonocoxites dorsally with apical large, setose, lobular projections. Gonostylus small, dorsal branch digitiform, tapering towards apex, with scattered small setae. Tergite 9 weakly sclerotized, wide, short, restricted to basal portion of terminalia. Parameres projecting slightly beyond gonocoxite apical margin. Aedeagus short. Cerci typically well developed, lobular, setose, projecting beyond distal margin of gonocoxites.

**Female.** Unknown.

##### Etymology.

The specific epithet of this species combines the Latin word *altus* (nominative, adjective masculine or neutre) for “high”, with the name *andina* (nominative, adjective feminine) for the South American mountain chain system, referring to the presence of this species in higher elevations in the Andean ecosystems.

#### Neuratelia colombiana sp. nov.

Taxon classificationAnimaliaDipteraMycetophilidae

http://zoobank.org/5A4A2D1B-CB31-4591-A8FF-075C58A0E2ED

Figs 2B , 3B,E, H , 4B , 5D–F , 6C, D

##### Type locality.

Colombia, department of Antioquia, Sonsón municipality, Norí municipal rural settlement, Norí mountain, paramo of Sonsón locality, 5°48.716'N, 75°16.1066'W, alt. 3045 m a.s.l. paramo, A. Cardona and D. Cardona leg.

##### Type specimen.

Holotype male, wing mounted in Euparal on microscope slide, rest of body in alcohol 96%. Original label: “Colombia, Antioquia, Sonsón, Vda. Norí, cerro Norí, páramo de Sonsón, 5°48'46.3"N, 75°16'6.398"W, páramo, 3045 m, 1–12 Sept. 2018. Proyecto Moscas de las flores. M. Salinas, A.M. Echeverry and A.L. Montoya cols. CEUA 94079”.

##### Material examined.

**Holotype** Colombia ♂, department of Antioquia, Sonsón municipality, Norí locality, Norí mountain, paramo of Sonsón; 5°48.7716'N, 75°16.1966'W, alt. 3045 m a.s.l.; 1–12 Sept. 2018. Proyecto Moscas de las flores. M. Salinas and A.L. Montoya leg.; paramo; Malaise trap; CEUA 94079. **Paratype** 1♂, Colombia, same data as holotype but differ on: 5°48.5751'N, 75°16.1178'W; alt. 2888 m a.s.l.; 7–9 May 2014; A. Cardona and D. Cardona leg.; forest; Malaise trap; CEUA 94076.

##### Diagnosis.

Thorax light brown, scutum with a pair of dark slender dorsocentral stripes. Vein CuA with gentle distal curve, CuP short, ending around mid of CuA. Syngonocoxite not extending to distal margin of terminalia; gonocoxites without an inner dorsal projection, inner apical projections small, slender, acute. Gonostylus ventral branch digitiform, dorsal branch short, pointed.

##### Description.

**Male** (Fig. 2B). Body length, 5.2 mm. ***Head*** (Fig. 3B). Width, 0.54mm, height, 0.49mm. Vertex brown, with abundant brownish short setae. Three ocelli, mid ocellus smaller; lateral ocelli separated from eye margin by less than their diameter. Occiput brown. Ommatotrichia abundant, short, yellowish. Scape and pedicel brownish yellow, cylindrical, scape longer than pedicel, both with small brownish-yellow setae; 14 flagellomeres, mostly light brown, with scattered dark small setae; first flagellomere almost twice as long as second. Frons and clypeus light brown, subrectangular, covered with dark setae; palpus with five light brown palpomeres, lighter towards apex, last palpomere about twice as long as fourth. ***Thorax*** (Figs 3E, H). Scutum light brown, with a pair of dark brown dorsocentral stripes and a weak medial dark stripe, all three connected at posterior margin of scutum, a pair of dark brown lateral longitudinal stripes above wings. A row of stronger setae above wing and a single row of differentiated dorsocentrals. Scutellum dark brown, with a lateral pair of scutellars on each side and three medial setae on posterior margin. Pleural sclerites dark brown, with some light areas on dorsal half of katepisternum and at dorsal end of mesepisternum. Pleural membrane yellowish brown. Antepronotum with nine long setae, proepisternum with four setae. Proepimeron, anepisternum, katepisternum, mesepimeron, and metepisternum bare. Laterotergite with 18 long dark setae, mediotergite with a row of dark setae on ventral half. Halter pedicel yellowish, knob light brown, both setose. ***Legs.*** Coxae and femora yellowish brown, tibiae and tarsi light brown. Foreleg tibiae with ventral oval depression with abundant irregular trichia; first tarsomere about 1.5 times as long as tibiae. Tibiae and tarsi with erect, short, dark setae along their entire length. Tibial spurs 1:2:2, brown, slightly longer than tibia width at apex. Tarsal claws with a large apical tooth and a smaller basal one. ***Wing*** (Fig. 4B). Length 4.0 mm, width 1.8 mm. Membrane light brown, densely covered with decumbent macrotrichia on all cells, veins brownish. Sc complete, setose ventrally, reaching C well beyond base of Rs, almost at mid length of wing; sc-r present, bare, placed basal to middle of Sc. C ending at apex of R_5_. R_1_ long, reaching C beyond distal fifth of wing. First sector of Rs oblique, setose ventrally, slightly longer than r-m. R_5_ sinuous, reaching C at wing apex, r-m bare, oblique. M_1+2_ stem shorter than medial fork. M_1_ weak, obsolete basally. M_4_ complete. CuA complete, gently curving on distal third. CuP short, reaching around level of mid CuA. ***Abdomen.*** Segments chestnut brown, cylindrical, slender, with long brownish setae on tergites and sternites. Sternite 8 not projecting posteriorly beyond basal half of gonocoxite. Tergite 8 well developed, with short medial projection covering less than basal half of tergite 9, almost rectangular, occupying anterior fourth of terminalia. ***Terminalia*** (Figs 5D–F, 6C, D). Light brown. Longer than wide. Gonocoxite ventral surface almost fused medially, forming a syngonocoxite with a deep ventral medial cleft, extending close to half the level of gonocoxite. Gonocoxites elongate, with a short inner dorsal projection, apically truncated, and a slender, dorsal-apical projection. Gonocoxal apodem conspicuous, with acute apex. Dorsal branch of gonostylus wider, tapering towards apex, ventral branch digitiform, with scattered setae; parameres curved, approaching each other medially and then diverging, slightly projecting beyond apex of gonocoxites. Aedeagus very short, not reaching apex of cerci. Cerci typically slender, digitiform, with abundant short strong setae, not projecting beyond distal margin of gonocoxites.

**Female.** Unknown.

##### Etymology.

The specific epithet *colombiana* (nominative, adjective feminine) of this species refers to Colombia, the country in which the type materials were collected.

#### Neuratelia elegans

Taxon classificationAnimaliaDipteraMycetophilidae

(Lane, 1948) comb. nov.

Figs 2E , 3C, F, I , 4C , 5G–I , 6E, F

Eudicrana elegans Lane 1948: 251, fig. 8 (gonocoxite and gonostyle), 9 (“mesosome”), 10 (tergite 9). **Type.** Holotype male, pinned, genitalia in permanent Canada balsam microslide mounting pinned with specimen. Original label: “Brazil, São Paulo, Salesópolis, Estação Biológica de Boraceia, xi.1947, F. Travassos and E. Rabello leg. MZUSP-07105”.
Neuratelia sapaici Lane 1952: 135, fig. 3 (male terminalia), syn. nov. **Type.** Holotype male, pinned, genitalia in permanent Canada balsam microslide mounting pinned with specimen. Original label: “Brazil, São Paulo, Salesópolis, Estação Biológica de Boraceia, 14 viii. 1947, E. Rabello, F. Travassos and J. Lane leg. MZUSP-04030”.

##### Type locality.

Brazil, state of São Paulo, Salesópolis municipality, Boraceia Biological Station [23°41.4378'S, 45°49.4288'W].

##### Material examined.

**Holotype** ♂ Brazil; State of São Paulo, Salesópolis municipality, Boraceia Biological Station; [23°41.4378'S, 45°49.4288'W]; Nov. 1947; F. Travassos and E. Rabello leg. MZUSP–07105. **Paratype** 1♂; Brazil, same data as holotype but differ on: 14 Aug.1947; E. Rabello, F. Travassos and J. Lane leg. MZUSP–04030.

##### Diagnosis.

Thorax brown, scutum without longitudinal stripes. Vein CuA with strong curve on distal third, CuP long, ending at about level of distal third of CuA. Gonocoxite fused medially only on basal half, with a ventral inward lobular distal projection and a digitiform laminar projection at dorsal surface of terminalia; gonostylus short, triangular in ventral view, with a flat basal projection dorsally.

##### Redescription.

**Male** (Fig. 2C). Body length 6.8 mm. ***Head*** (Fig. 3D) Width 0.51mm, length 0.54mm. Vertex dark brown, with abundant brownish setae. Three ocelli, mid ocellus smaller; lateral ocelli separated from eye margin by less than their diameter. Occiput dark brown. Ommatotrichia abundant, short, yellowish. Scape and pedicel yellowish brown, cylindrical, scape longer than pedicel, slightly darker, both with short setae; with 14 dark brown flagellomeres, with scattered yellowish setae, first flagellomere almost twice as long as second flagellomere. Frons and clypeus brown, subrectangular, densely covered with yellowish setae; labella caramel brown, with five light brown palpomeres, last palpomere about twice length of fourth. ***Thorax*** (Figs 3F, I). Scutum, scutellum, and pleura brownish, scutum with two darker stripes connected medially at posterior end and reaching scutellum, with scattered yellowish setae, a single row of differentiated dorsocentrals, and no clear row of acrostichals; prealars and postalars strong. Scutellum brownish, with two stronger setae laterally and two medially, and scattered, smaller, marginal setae. Pleural sclerites mostly brown, ventral half of katepisternum and mediotergite dark brown; pleural membrane yellowish brown. Proepimeron, anepisternum, katepisternum, mesepimeron, and metepisternum bare, antepronotum and proepisternum setose, laterotergite with 25 well developed brown setae, mediotergite with a row of around 25 well-developed setae across ventral margin. Halter setose, pedicel yellowish, knob light brown. ***Legs.*** Coxae yellowish brown, darker apically, femora light brown, tibiae and tarsi brown. Fore femora, tibiae, and tarsi missing in the holotype; mid tibia with dorsal and ventral irregular rows of slightly longer dark setae, hind tibia with a regular row of dark setae posteriorly; mid tibia spurs subequal, almost twice apical width of tibia, hind tibia outer spur longer than inner spur. Mid and hind first tarsomeres very long (distal tarsomeres of mid and hind tarsi missing in holotype). ***Wing*** (Fig. 4C). Length, 4.6 mm, width, 1.5 mm. Membrane light brown, without dark maculae, membrane densely covered with microtrichia and decumbent macrotrichia on all cells. Anterior veins brown, medial and cubital veins yellowish brown; Sc complete, reaching C well beyond base of Rs, almost at the middle of anterior margin of wing, setose; sc- r present basally, well before origin of Rs, bare. C ending at apex of R_5_. R_1_ long, reaching C beyond distal fifth of wing. First sector of Rs oblique, bare, only slightly longer than r-m. R_5_ slightly sinuous, reaching C near wing apex. Vein r-m oblique, bare. M_1+2_ stem more than twice length of r-m. M_1_ obsolete basally. Medial and cubital veins complete, reaching wing margin. CuA with a strong curve towards base at distal third. CuP well sclerotized, extending to level of distal third of CuA. ***Abdomen.*** Brown, cylindrical, slender, with long dark setae covering tergites and sternites. Tergite 8 longer than wide, projected medially, sternite 8 wider than long, with a medial projection apically. ***Terminalia*** (Figs 5G–I, 6E, F). Syngonocoxite extending medially slightly beyond half the length of the gonocoxite; gonocoxites with a pair of inward lobes ventrally, with setulae, and a dorsoapical lobe slightly projecting distally beyond the base of the gonostyli; gonocoxites with a flat digitiform inner projection dorsally. Gonostylus relatively simple, covered only with setulae, without spines or long setae, ventral branch triangular in ventral view, dorsal branch short, flat. Tergite 9 weakly sclerotized, restricted to basal half of terminalia, with a group of short setae at each side. Parameres straight (not curved as in many species of the genus), reaching level of gonocoxite apex distally. Aedeagus elongate, straight. Sternite 10 present as a pair of elongated sclerotized stripes with setae. Cerci typicallywell-developed, lobular, extending distally almost as far as the apex of gonostylus, touching each other medially, but without evidence of fusion.

**Female.** Unknown.

##### Comments.

The holotypes of *Eudicranaelegans* and *Neurateliasapaici* are males and originally had their terminalia slide-mounted between cover slips, pinned with the respective specimens. The terminalia of both species are identical in every aspect and as well as the general colour of the specimens. Lane’s (1948, fig. 8) illustration of the gonocoxite and the gonostyle of *E.elegans* makes clear its identity with Lane’s (1952, fig. 3) illustration of the male terminalia of *N.sapaici* (Figs 5G–I, 6E,F). Along the original description of *Eudicranaelegans*, Lane (1948: 252) mentioned the interrupted M_1_, but did not comment on the lack of R_4_, which would immediately raise suspicious of the generic position of the species. We propose a new combination of the *Eudicranaelegans* and the synonymy of *Neurateliasapaici*.

## Discussion

The distinction between the three known Neotropical species of *Neuratelia* is very straightforward based on thorax coloration, specifically the patterns of stripes over the scutum, but the wing venation, morphology of clypeus, and the male terminalia are also distinctive. *Neurateliaaltoandina* shares with *N.elegans*, some distinctive characters, such as the length of CuP, while in *N.colombiana* CuP is shorter, extending beyond mid of CuA length. Body coloration of *N.colombiana* is lighter, with yellowish brown and brown tones; and a the scutum has a darker V-shaped brown mark over a lighter, ochre-brown background color, while *N.altoandina* and *N.elegans*, have a more homogeneously brown scutum. *Neurateliaaltoandina* and *N.elegans* have a rather strong curve on the distal third of CuA, reaching the wing margin at an angle of about 90°, while *N.colombiana* has a gentle distal curve, reaching the wing margin at an acute angle. Also, *N.altoandina* and *N.elegans* have a slightly longer CuP than *N.colombiana*. *N.colombiana* and *N.elegans* share a subrectangular shape of the clypeus, while the clypeus in *N.altoandina* is subtriangular.

The male terminalia is also distinct between the three species. In *N.altoandina* and *N.elegans* the male terminalia is wider than long, while in *N.colombiana* it is longer than wide. The ventral branch of the gonostylus is nearly digitiform in *N.altoandina* and *N.colombiana*, while in *N.elegans* it is triangular in ventral view. In *N.elegans*, the gonostylus dorsal branch is a flat, short lobe. The distal margin of the syngonocoxite in *N.altoandina* extends medially almost to the level of the distal end of the gonocoxite, while in *N.colombiana* it is slightly shorter, and in *N.elegans* the syngonocoxite extends to only half the length of the gonocoxites. The gonocoxites in *N.altoandina* have lobular dorsal projections distally, while in *N.colombiana* there is a slender acute projection and in *N.elegans* a digitiform flat projection. In *N.elegans*, the gonocoxites have an additional ventral lobe distally, which projects medially. Finally, *N.altoandina* and *N.elegans* have a pair of very characteristic lobular cerci, different from the slender, much short cerci in *N.colombiana*.

All *Neuratelia* species have a relatively short tergite 9, restricted to the anterodorsal portion of the male terminalia, in such a way that the gonocoxites project well beyond the distal margin of tergite 9 (e.g. Matile 1974: figs 2–7; Zaitzev 1994: figs 5, 6; Sasakawa 2004: figs 1–5; Kurina et al. 2015: figs 7, 21, 22). In some species, tergite 9 extends only half way the extension of the gonocoxite, but in other species it is restricted to an even more anterior position. The syngonocoxite medial extension, in most cases, reaches two-thirds of the length of the gonocoxite medially. Thus, the condition in *N.altoandina* is quite extreme, with the syngonocoxites covering the entire ventral surface of the terminalia. The shape of the gonostylus is variable between the species and in some cases very complex. Some *Neuratelia* species have a simpler gonostylus, as in *N.spinosa* Matile (Matile 1974: fig. 4), but it can also be very complex, as in *N.microdigitata* Sasakawa (Sasakawa 2004: fig. 4), *N.jabalmoussae* Kurina et al., *N.caucasica* Zaitzev, and *N.minor* (Lundström) (Kurina et al. 2015: figs 9, 11, 13). The parameres are usually curved, close to each other midway to apex and then strongly diverging distally.

Publications on *Neuratelia* do not provide a formal discussion on species groups or on the relationships within the genus. The only exception is Kurina et al. (2015), who indicated two pairs of species, *N.jabalmoussae*/*N.caucasica* and *N.nemoralis*/*N.salmelai*, based on a study with morphological and molecular data.

It is not possible to discuss the placement of the Neotropical species of *Neuratelia* within the genus without a broader phylogenetic study involving all species of the genus. It is clear, however, that the three Neotropical species share a clearly apomorphic feature: the gonocoxite internal dorsal projection. This feature is absent in all other species of the genus illustrated in the literature and suggests that this Neotropical group may be a monophyletic clade within the genus. *Neurateliacolombiana* and *N.elegans* might form together a clade, as the wing features (particularly the strong distal curve on vein CuP, quite unique in the genus) clearly suggest. The Nearctic and Palaearctic species of the genus with wing illustrations (e.g. *N.sayi* in Vockeroth 1981: fig. 36; *N.nemoralis* Meigen in Matile 1974: fig. 1) show a more strongly sinuous R_5_, a condition not so distinct in the Neotropical species. In this sense, the relatively simple gonostylus and the less sinuous R_5_ in the Neotropical species of *Neuratelia* may correspond to plesiomorphic conditions, indicating that the set of characters present in the Nearctic and Palearctic species may reflect the existence of a large Holarctic clade in the genus.

*Neuratelia* is currently unknown from Chile, but it is likely that the genus is also present there. This would confirm another case of a southern temperate South America group (with representatives in Chile, southern Argentina, and southern Brazil) that extends its distribution the north of the Andes, reaching of Colombia. This pattern is also known, within the Mycetophilidae, in the genera *Paraleia* (Oliveira and Amorim 2012), *Duretophragma* Borkent, *Eudicrana* Loew, and others, and in the Rangomaramidae clade with the genera *Chiletricha* and *Eratomyia* (Amorim and Rindal 2007; Amorim and Falaschi 2010).

## Supplementary Material

XML Treatment for Neuratelia

XML Treatment for Neuratelia altoandina

XML Treatment for Neuratelia colombiana

XML Treatment for Neuratelia elegans

## Supplementary materials

Supplementary material 1 Neuratelia Neotropical species list Data type: species data This dataset is made available under the Open Database License (http://opendatacommons.org/licenses/odbl/1.0/). The Open Database License (ODbL) is a license agreement intended to allow users to freely share, modify, and use this Dataset while maintaining this same freedom for others, provided that the original source and author(s) are credited.Carolina Henao-Sepúlveda, Marta Wolff, Dalton de Souza Amorim
